# Effectiveness of the live attenuated rotavirus vaccine produced by a domestic manufacturer in China studied using a population-based case–control design

**DOI:** 10.1038/emi.2015.64

**Published:** 2015-10-28

**Authors:** Shan-Shan Zhen, Yue Li, Song-Mei Wang, Xin-Jiang Zhang, Zhi-Yong Hao, Ying Chen, Dan Wang, Yan-Hong Zhang, Zhi-Yong Zhang, Jing-Chen Ma, Peng Zhou, Zhen Zhang, Zhi-Wei Jiang, Yu-Liang Zhao, Xuan-Yi Wang

**Affiliations:** 1 Key Laboratory Medical Molecular Virology, MoE/MoH, and the Institutes of Biomedical Sciences, Shanghai Medical College, Fudan University, Shanghai 200032, China; 2 Laboratory of Molecular Biology, Training Center of Medical Experiments, School of Basic Medical Sciences, Fudan University, Shanghai 200032, China; 3 Zhengding County Center for Disease Control and Prevention, Zhengding 050800, Hebei Province, China; 4 Hebei Province Center for Disease Control and Prevention, Shijiazhuang 050800, Hebei Province, China; 5 Department of Health Statistics, Fourth Military Medical University, Xi'an 710032, Shanxi Province, China

**Keywords:** attenuated vaccine, case–control study, China, effectiveness, rotavirus

## Abstract

A universal rotavirus (RV) immunization program is a potentially cost-effective measure for preventing RV infection in China. However, the efficacy of the only licensed RV vaccine (Lanzhou lamb rotavirus vaccine, LLR), which is made by a domestic manufacturer, has not been proven by a properly designed clinical trial. In October 2011 to March 2012, to measure the potential protection provided by LLR, a case–control study nested in a population-based active diarrhea surveillance study of children <5 years of age was conducted in rural Zhengding county. During the study period, 308 episodes of diarrhea were identified as being caused by RV infection, resulting in an incidence rate of 48.0/1000 people/year. The predominant RV serotype was G3 (61.5%), followed by G1 (15.2%), and G9 (6.5%). Overall, a protection of 35.0% (95% confidence interval (CI), 13.0%–52.0%) was identified, and higher protection was found among moderate RV gastroenteritis cases caused by the serotype G3 (52.0% 95% CI: 2.0%–76.1%). A concurrently conducted case–control study comparing non-RV viral diarrheal cases with non-diarrheal controls in the same population found that the RV vaccine offered no protection against non-RV diarrhea. Even under a less ideal immunization schedule, the oral LLR conferred a certain level of protection against RV gastroenteritis. However, further studies are needed to understand the full characteristics of the LLR, including its efficacy when administered following the optimal regimen, the potential risk of inducing intussusception, and the direct and indirect protective effects of LLR.

## Introduction

Acute gastroenteritis is the leading cause of childhood illness worldwide and in China.^1,2^ Rotavirus (RV) is the leading pathogen that causes severe gastroenteritis in children,^3,4,5^ and infects virtually all children by 5 years of age in both industrialized and developing countries.^4,6^ Improving the safety control of water and food and implementing a better sanitation program seem unlikely to reduce the occurrence of diseases caused by RV.^7^ In China, published data indicates that RV-associated hospitalizations account for 32%–50% of all hospitalizations for diarrhea among infants and children <5 years of age.^8,9,10,11^ With the introduction of effective RV vaccines produced by major Western pharmaceutical companies, a cost-effectiveness analysis indicated that a universal RV immunization program can be expected to result in high net savings by decreasing the hospitalizations of immunized patients.^6^ As China is the country with the largest human population in the world, it should also consider RV vaccination as a potential cost-effective measure against RV infection.^4^

A RV vaccine called Lanzhou lamb rotavirus vaccine (LLR) has been developed and licensed in China since 2000.^4,12^ At the end of 2014, a total of 60 million doses of LLR had been distributed to children in China. However, the efficacy of LLR has not been recognized internationally as it has not been confirmed by a properly designed pre-licensure clinical trial.^12,13^ Several hospital-based case–control studies have measured the effectiveness of LLR in the past; however, the results of those studies were inconsistent.^14,15,16^ The current report describes a population-based active surveillance study conducted between 1 October 2011 and 31 March 2012 among children less than 5 years of age in Zhengding county, Hebei Province, China, which is located 270 km south of Beijing. The aim of this study was to define the occurrences of RV, calicivirus, astrovirus, and adenovirus infection in this study population, and this study provided a good opportunity for evaluating the effectiveness of LLR using a population-based case–control design.

## Materials and methods

### Study population and information collection

In total, 34 villages located in five townships in Zhengding County, Hebei Province, China, were selected as the catchment area for this population- and health-care facility-based viral diarrhea surveillance targeting children <5 years of age. All health-care providers possibly offering health care for children living in the catchment area were included in the surveillance system, including 101 village clinics, five township hospitals, and one county hospital. According to the registration records from the EPI registration system in Zhengding County's Center for Disease Control and Prevention (Zhengding CDC), in 2011, 5724 children less than five years of age lived in the catchment area.

### Surveillance design

The surveillance was conducted in a six-month period from 1 October 2011 to 31 March 2012, which covered the entire peak season for RV diarrheal illness in children.^8,10,11^ The study protocol was developed using the generic protocol for RV surveillance from the World Health Organization.^17,18^ The study population included children less than five years of age residing in the catchment area who presented to a participating health-care facility with acute–onset diarrhea and whose parents or guardians gave informed consent for enrollment in this study. A trained community health-care worker visited each child living in the corresponding community (either at home or over the telephone) once every week to collect diarrhea information independently from that obtained by the health-care facility.

For every patient presenting with diarrhea, a case report form (CRF) was generated describing demographics, medical history, physical examination, and the management plan. Two rectal/stool swabs or a stool specimen were obtained. A community health-care worker visited the patients once a week until they had full recovered from diarrhea. At these follow-up visits, a questionnaire was completed that recorded demographics, medical history since the onset of diarrhea, follow-up examination, and management practices executed.

Bulk stools were obtained by health-care providers within 1 h of presentation and were tested with a commercial enzyme-linked immunosorbent assay (ELISA) (DAKO Diagnostics Ltd, Cambridgeshire, UK) according to the manufacturer's instructions. Stool samples that were positive for RV were G and P genotyped using a heminested multiplex reverse-transcriptase polymerase chain reaction (RT-PCR) assay.^19^ In addition, all stool samples, regardless of the results of RV detection, were tested for calicivirus and astrovirus using RT-PCR and for adenovirus using PCR as previously reported.^20^ Diarrhea was defined as three or more loose bowel movements during a 24-h period. Recovery was defined as the cessation of loose stools for three consecutive days. A RV episode was defined as an episode of diarrhea with a positive RV ELISA test.

To quantify the severity of gastroenteritis, a modified version of the widely used Vesikari Clinical Severity Scoring System was used, with scores ranging from 0 to 20. In China, an intravenous infusion (IV) therapy is widely used upon the presentation of diarrhea in health-care facilities, reducing the incidence of dehydration in clinical practice; thus, the parameter named dehydration was defined as zero in this analysis. Additionally, due to the reimbursement policy of the health insurance system in China, a higher percentage of medical expenses incurred during hospitalization are covered by insurance than expenses incurred as an outpatient, resulting in hospitalization being an unreliable indicator of disease severity. In this analysis, hospitalization was defined as receiving IV therapy ≥ three consecutive days at a health-care facility. An episode of gastroenteritis was considered severe with a score ≥11 and moderate with a score ≥7 and <11.^21^

### Lanzhou lamb rotavirus vaccine

Currently, the only licensed LLR in China was developed by Lanzhou Institute of Biological Products Co. Ltd. in 2000.^4,12^ The vaccine strain was originally isolated from a lamb with diarrhea in 1985 and is characterized as G10P[12]. The attenuation of this strain was completed by passaging it in primary kidney cells of a newborn calf for 37 passages, named LLR-85-37.^22^ The vaccine was reported to be safe and immunogenic;^23^ however, its efficacy has not been proven by a well-designed, randomized, placebo-controlled trial.^12,13^ The LLR is a liquid formulation with buffer containing >5.5 log CCID_50_ (50% cell culture infective dose) per dose in a volume of 3 mL, and it is given to children between 2 and 35 months of age at one dose per year for three consecutive years. The LLR was introduced in 2008 and has reached a coverage of ∼30% among children less than five years of age in the study area.

### Study design for vaccine effectiveness

Two case–control studies nested within the population-based surveillance were applied to measure the effectiveness of the LLR. The first (effectiveness study) aimed to estimate the protective effectiveness of the LLR^24^ and the second (bias indicator study) aimed to assess whether the results of the effectiveness study could be attributed to bias.^25^ The source population for cases and controls in each of the two studies was nested in the viral diarrhea surveillance. The cohort was dynamic and included children who were still less than 60 months of age on 31 March 2012, which was the date of study completion. Because RV infection in children is not affected by socio-economic status, to minimize potential selection bias, the effectiveness study was performed using a matched case–control design. The first study contrasted cases of RV diarrhea with non-diarrheal controls; the second study contrasted astrovirus, adenovirus, and calicivirus diarrhea cases with non-diarrheal controls. The absence of vaccine protection in the second study was considered to suggest the absence of bias in the first analysis.

#### *Definition and selection of cases and controls*

**Figure 1:**
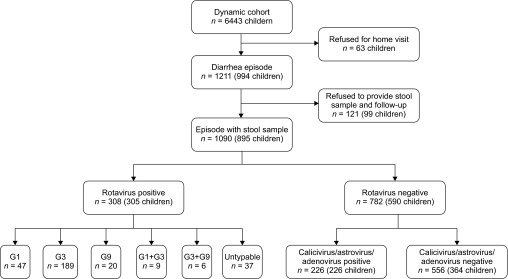
Summary of participants in the two case–control studies. Note, among RV-positive cases, 37 cases were co-infected with calicivirus, astrovirus, and/or adenovirus.

In the effectiveness study, RV diarrhea was defined as a laboratory-confirmed RV infection in a child less than five years of age. We restricted cases for the bias indicator study to those presenting with astrovirus, adenovirus, or calicivirus diarrhea. Repeated episodes during the study period were categorized by the following principles: (i) patients with RV-, calicivirus-, astrovirus-, and adenovirus-negative episodes were excluded from both analyses; (ii) for patients with repeated RV infections, only the first-identified episode was included in the effectiveness study (there were only three reinfections with RV); (iii) diarrhea episodes in patients positive for RV and calicivirus, astrovirus, or adenovirus were classified as RV diarrhea; (iv) patients with calicivirus, astrovirus, or adenovirus diarrhea were included as a case only once in the bias indicator study. The assembly of the two sets of cases is shown in Figure 1.

During the study period, children less than five years of age without diarrhea who lived in the catchment area were candidates for controls for both the effectiveness and bias indicator studies. For each RV case and non-RV case (calicivirus, and/or astrovirus, and/or adenovirus), four individually matched controls were selected in order of identification number (ID) assigned in the census database based on the following criteria: (i) living in the same township as the case; (ii) same gender as the case; and (iii) born within 90 days of the case. No control could be shared within or between the effectiveness study and bias indicator study. Considering the relatively wide age-matching caused by the one child policy, a sensitive analysis applying different age-matching was performed to adjust for potential bias.

#### *Ascertainment of vaccination and potentially confounding variables*

Because the LLR has not been integrated into the Expanded Program on Immunizations (EPI) program, the parents of vaccinees have to pay for it out of pocket. Based on the current rule, the producers of non-EPI vaccines should compensate individuals for medical costs incurred due to vaccination-associated adverse events. The sole evidence for compensation is the record on the immunization card possessed by the parents. Before launching surveillance, LLR status was copied from vaccination cards held by parents or guardians and entered into a census database during the home visit for the census survey. Each qualified child was assigned an ID number. Vaccination was defined as receipt of at least one dose of the LLR documented on the vaccination card, and most vaccinees only received the first dose of the LLR. For those individuals who were not able to show the immunization card, a sensitive analysis of vaccine effectiveness was conducted in regards to the different classifications of vaccination. A breakthrough RV infection was defined as a laboratory-confirmed RV infection in a vaccinated child at least 14 days after completion of the first dose of the LLR.

### Data management and analysis

All CRFs were double entered into a custom-made data entry program (the EpiData program, version 3.1). The data management programs include error as well as consistency check programs. We used the SAS program (SAS Institute Inc., Cary, NC, USA) for the statistical analyses. A total of 288 RV infection cases were required to compute a vaccine effectiveness of 70% with a statistical power of 80% using a matched design with a case to control ratio of 1:4. The incidence rates were calculated based on the cohort residing in the catchment area during the study period. Before launching the study, demographic data were transferred from the EPI program system stored in Zhengding CDC to the census database and were verified during the census survey. An ID was assigned to each child by township following the sequence of registration in the EPI system. After the completion of the study and data cleaning, the census database containing vaccination histories was linked to the surveillance database, which included clinical and laboratory information by ID and child name. For both the effectiveness and bias indicator studies, vaccine protections against RV gastroenteritis and, specifically, severe RV gastroenteritis were calculated using a conditional logistic regression model. Vaccine protection, expressed as (1 minus the adjusted odds ratio of RV gastroenteritis in vaccinees versus non-vaccinees) × 100%, was estimated by exponentiating the coefficient for the vaccine variable in the models.^24,26^ All *P* values and 95% confidence intervals (95% CI) were interpreted in a two-tailed fashion. Statistical significance was designated as a *P* value less than 0.05.

### Ethics

This study was reviewed and approved by the Institutional Review Board of the Hebei Center for Disease Control and Prevention, and the Institutional Review Board of the Institutes of Biomedical Sciences, Fudan University. Written inform consent was obtained from the parent/guardian of each child. The study was performed in accordance with the ethical standards laid down in the 1964 Declaration of Helsinki and its later amendments.

## Results

During the study period, the total number of children <5 years of age enrolled in the dynamic cohort was 6441 (5533 and 5733 children were <5 years of age at the time points of 1 October 2011 and 31 March 2012, respectively). Except for 77 (1.2%) children who emigrated during the study period, and thus excluded in this analysis, vaccination status could not be determined for 46 (0.7%) children whose vaccination card was lost. Finally, 1412 (21.9%) children were confirmed to have received at least one LLR dose, with a cumulative coverage of 22.3% (1412/6441). The majority of children (90.5%) received the first LLR dose between 6 months and 24 months of age (Table 1).

From 1 October 2011 to 31 March 2012, 1211 diarrhea episodes were reported through the surveillance system (Figure 1). Of these, 1091 (90.1%) provided stool samples for a RV test, and 308 episodes were RV-positive, resulting in an incidence rate of 48.0/1000 people/year. The predominant serotype of RV was G3 (61.5%), followed by G1 (15.2%), and G9 (6.5%). Of the RV-negative episodes, 226 were confirmed as calicivirus, astrovirus and/or adenovirus infection, and they composed the non-RV case group for the bias indicator study. Overall, 58 episodes of severe gastroenteritis were observed during the entire study period. More episodes of severe and moderate gastroenteritis were found among RV gastroenteritis cases (13.3% and 34.7%) than among non-RV gastroenteritis cases (2.2% and 20.0%) (*P* < 0.0001).

Overall, 305 RV cases (mean age: 14.9 months with a standard deviation (SD) of 7.7 months) and 1220 controls (mean age: 17.6 months with a SD of 7.7 months) were included in the effectiveness study, and a protection rate of 35.0% (95% CI, 13.0%–52.0%) was identified, with the children whose vaccination cards were unavailable being considered to have not received the LLR. Higher protection was found against moderate/severe RV gastroenteritis caused by serotype G3 (53.0% 95% CI: 15.0%–75.0%). Because the majority of RV gastroenteritis cases were caused by the G3 serotype during the study period, a sub-group analysis was performed to estimate the protection against any G3 serotype RV gastroenteritis and, specifically severe G3 RV gastroenteritis; other sub-group analyses were not performed due to the small sample size. A notable difference in vaccine protection was not detected between conservative and non-conservative scenarios (Table 2). Based on the conservative scenario, the average ages of RV attack were 17.8 months (SD: 7.9 months) and 12.1 months (SD: 7.2 months) among children who did and did not receive the vaccine, respectively (*P* < 0.001). For patients who developed RV diarrhea after receipt of the LLR, the median time between vaccination and presentation of RV diarrhea was 7.4 months [interquartile ratios (IQR): 4–12 months].

The bias indicator study included 226 children with gastroenteritis caused by calicivirus, astrovirus and/or adenovirus and 904 controls. The mean ages of the non-RV cases and controls were 16.8 months (SD: 10.5 months) and 18.3 months (SD: 10.5 months), respectively. The protection against RV gastroenteritis was estimated to be 12.5% (95% CI: − 20.4% to 36.5% *P* = 0.41).

To detect potential bias introduced due to relatively wide age-matching, odds ratios were calculated by the subsets of controls born within 60 and 45 days of the case's date of birth and were compared to the results obtained by matching the controls to within 90 days of age of the cases based on the conservative scenario (Table 3). Fortunately, a notable difference was not observed with the narrowing of the matching age.

## Discussion

In early 1980s, Vesikari was the first to attempt the clinical development of an oral vaccine derived from a bovine strain of RV. The oral bovine vaccine showed 50% protection against any RV diarrhea and 88% protection against severe RV diarrhea. This result indicated that live oral RV vaccines could be more highly effective against severe RV diarrhea than against milder disease.^27^ Subsequently, several RV vaccines were developed worldwide.^12,28^ Of these, the LLR was the second to be developed and was licensed in 2000 in China. However, to date, few data are available about the efficacy of the LLR due to the lack of a proper phase III clinical trial.^12,13^ While not ideal, the population-based diarrhea surveillance provided a unique opportunity to test the effectiveness of the LLR in a real-world setting. Overall, a low-level of protection against RV gastroenteritis, regardless of severity, was demonstrated by this current case–control study nested in an active population-based surveillance. Similar to previously published studies,^29,30^ higher protection was found for severe/moderate than for mild RV gastroenteritis. It must be admitted that even the highest protection of 52% against moderate RV gastroenteritis caused by serotype G3 is not satisfactory for a prophylactic vaccine, because the current used regimen for LLR, which children receiving one dose per year for three consecutive years between the ages of 2 and 35 months, is generally not preferred. Worldwide, the consensus for optimized immunization schedules to maximize the efficacy of a RV vaccine is to vaccinate before RV gastroenteritis occurs and before a sizeable proportion of the target population acquires natural infection, which is typically at 6 months of age.^5,31^ Considering the typical age distribution of RV gastroenteritis, RV vaccination of children >24 months of age is not recommended.^6^ Conversely, in the current study, only 1.3% of children received the first LLR dose before 6 months of age. This may have resulted in a great reduction of the real protection against RV gastroenteritis offered by LLR immunization. Moreover, in contrast to randomized, controlled clinical trials (RCTs), which are idealized evaluations of vaccine efficacy, the present study was conducted under the real-life conditions of a routine public health program and, thus, measured vaccine effectiveness.^32^ In recent years, RCTs conducted in Asian and Africa countries observed inferior efficacies of two internationally available RV vaccines, namely RotaRix and RotaTeq.^33,34,35^ Taking all of this into consideration, studies aimed at measuring either the efficacy or effectiveness of LLR with a rationale regimen are strongly recommended.

Currently, the need for RV vaccines to induce serotype-specific protection in order to achieve promising protection against RV infection across regions and countries is not fully understood. Some studies have reported a dominance of serotype-specific neutralizing antibodies following natural infection,^36^ while other studies of naturally infected children found that the correlation of protection with neutralizing antibody titers was not serotype-specific.^37^ In the current study, though the LLR was animal sourced and characterized as G10P[12], it appeared to confer cross-protection against infection caused by RV serotype G3. Nevertheless, given the diversity and shifting of dominant RV subtypes in China, more studies, including in-depth analyses of serum antibody cross-reactivity, are needed.^38^

We are aware of several limitations pertaining to the present study. First, the use of an observational study design rather than a randomized trial is associated with potential bias. However, several features of this study helped to ensure the validity of the results. The study was nested within a population-based, prospective and active surveillance for diarrhea among children <5 years of age, and the controls were selected in a matched fashion from the same population, which likely reduced the selection bias. Although the histories of vaccination were documented retrospectively, they were collected prior to the surveillance without knowledge of case–control status. For the children without vaccination cards, a notable difference was not detected by sensitive analysis, regardless of the classification of vaccination status. Thus, the conclusions of this study were less likely to be weakened by misclassification bias.^39,40,41^ A similar likelihood of receiving or not receiving the vaccine is also critical for determining vaccine effectiveness. Though the willingness to pay for LLR in the catchment area was not assessed, several published studies concluded that the most important factor impacting non-EPI vaccine coverage was the cost of the vaccine.^42,43,44^ For RV infection, it is well-known that similar incidences are observed in both developing and developed countries. Thus, the potential economic inequality between families may not have biased the results. Moreover, a bias-indicator case–control study was performed using procedures identical to those for the study of vaccine protection. As expected, protection against diarrhea caused by calicivirus, astrovirus, and adenovirus was not found to be provided by LLR in any capacity.

Second, the inequality of probability to infection between cases and controls is critical for the evaluation of vaccine effectiveness using a case–control design. With regard to the epidemiologic characteristics of RV infection, children at different months of age are associated with different risks. Ideally, more strict age matching, for example, within 30 days, could be applied. However, due to the one-child policy in China, such strict matching criteria cannot reasonably be carried out in the real world. Fortunately, a notable difference in effectiveness was not observed in the sensitive analysis, which narrowed the matching age. Thus, there is no evidence to speculate that the current age matching biased the results.

Third, the number of laboratory-confirmed RV infections was not sufficient to thoroughly demonstrate the cross-protection against RV gastroenteritis caused by serotypes G1 and G9, which are the most common serotypes, and serotype G3 in China.^38^

In summary, even under a far-from-ideal immunization schedule, the oral LLR produced by a domestic manufacturer did confer a certain amount of protection against RV gastroenteritis in Zhengding county, which had a high incidence of RV infection during the study period.^4,5^ To thoroughly define the characteristics of the LLR, many factors, including its true efficacy with a rationale regimen, the potential risk of inducing intussusception, and its direct and indirect protective effects, need to be studied further.

## Figures and Tables

**Table 1:** Details of vaccination status among children who received at least one dose of LLR

Age at vaccination (month)	Number of children (%)		
	First dose	Second dose	Third dose
0–	17 (1.3)	0	0
6–	642 (47.2)	0	0
12–	342 (25.2)	6 (3.4)	0
18–	256 (18.1)	83 (46.6)	0
24–	104 (7.7)	54 (30.3)	1 (8.3)
30–	38 (2.8)	23 (12.9)	8 (66.7)
36–	13 (1.0)	12 (6.8)	3 (25.0)
Total	1412	178	12

**Table 2:** Estimates of the odds ratio (OR) for vaccination with human rotavirus vaccine (HRV) in the effectiveness and bias indicator studies

	Proportion vaccinated (%)			
	Case patients	Control subjects	OR (95% CI)	*P* value
**Effectiveness study: non-conservative scenario**				
*All severities*				
Against all serotypes	51/305 (16.7%)	304/1220 (24.9%)	0.65 (0.48–0.87)	0.0062
Against G3	31/204 (15.2%)	324/1321 (24.5%)	0.59 (0.40–0.87)	0.0076
*Moderate/severe illness*				
Against all serotypes	23/147 (15.7%)	332/1378 (24.1%)	0.61 (0.39–0.95)	0.0305
Against G3	12/100 (12.0%)	343/1425 (24.1%)	0.47 (0.25–0.85)	0.0136
**Effectiveness study: conservative scenario**				
*All severities*				
Against all serotypes	53/305 (17.4%)	305/1220 (25.0%)	0.68 (0.50–0.92)	0.0111
Against G3	33/204 (16.2%)	325/1321 (24.6%)	0.63 (0.43–0.92)	0.0162
*Moderate/severe illness*				
Against all serotypes	24/147 (16.3%)	334/1378 (24.2%)	0.64 (0.41–0.99)	0.0448
Against G3	13/100 (13.0%)	345/1425 (24.2%)	0.50 (0.28–0.91)	0.0224
**Bias indicator study: non-conservative scenario**				
All severities	49/226 (21.7%)	224/904 (24.8%)	0.86 (0.63–1.19)	0.3749
**Bias indicator study: conservative scenario**				
All severities	50/226 (22.1%)	226/904 (25.0%)	0.88 (0.64–1.20)	0.4122

Non-conservative scenario: vaccination history of those children who could not provide vaccination card was defined as having not received LLR; Conservative scenario: vaccination history of those children who could not provide vaccination card was defined as having received LLR.

**Table 3:** Sensitive analysis of VE against all serotypes of RV with different age matching based on the conservative scenario

	Proportion vaccinated (%)		
	Case patients	Control subjects	OR (95% CI)
**Matching age <90 days between cases and controls**			
All severities	53/305 (17.4%)	305/1220 (25.0%)	0.68 (0.50–0.92)
Moderate/severe illness	24/147 (16.3%)	334/1378 (24.2%)	0.64 (0.41–0.99)
**Matching age <60 days between cases and controls**			
All severities	53/305 (3.49%)	299/1214 (19.68%)	0.70 (0.52, 0.94)
Moderate/severe illness	17/109 (1.12%)	335/1410 (22.05%)	0.61 (0.36–1.03)
**Matching age <45 days between cases and controls**			
All severities	53/305 (3.50%)	294/1209 (19.42%)	0.71 (0.52–0.96)
Moderate/severe illness	17/109 (1.12%)	330/1405 (21.80%)	0.62 (0.37–1.04)

## References

[bib1] 1Wang XY, Du L, Von Seidlein L et al. Occurrence of shigellosis in the young and elderly in rural China: results of a 12-month population-based surveillance study. Am J Trop Med Hyg 2005; 73: 416–422. 16103614

[bib2] 2Yu WL. [Survey on the prevention and treatment of diarrhoea in part of regions, China.] Zhonghua Liu Xing Bing Xue Za Zhi 1989; 10: 257–260. Chinese. 2611865

[bib3] 3Liu N, Xu Z, Li D et al. Update on the disease burden and circulating strains of rotavirus in China: a systematic review and meta-analysis. Vaccine 2014; 32: 4369–4375. 24958704 10.1016/j.vaccine.2014.06.018

[bib4] 4Wang XY, Riewpaiboon A, von Seidlein L et al. The potential cost-effectiveness of a rotavirus immunization program in rural China. Clin Infect Dis 2009; 49: 1202–1210. 19739973 10.1086/605632

[bib5] 5Wang XY, Xu ZY, von Seidlein L et al. Incidence of diarrhea caused by rotavirus infections in rural Zhengding, China: prospective, population-based surveillance. J Infect Dis 2005; 192: S100–105. 16088791 10.1086/431507

[bib6] 6Rotavirus vaccines. WHO position paper – January 2013. Wkly Epidemiol Rec 2013; 88: 49–64. 23424730

[bib7] 7Parashar UD, Bresee JS, Gentsch JR et al. Rotavirus. Emerg Infect Dis 1998; 4: 561–570. 9866732 10.3201/eid0404.980406 PMC2640254

[bib8] 8Fang ZY, Wang B, Kilgore PE et al. Sentinel hospital surveillance for rotavirus diarrhea in the People's Republic of China, August 2001–July 2003. J Infect Dis 2005; 192: s94–99. 16088812 10.1086/431505

[bib9] 9Orenstein EW, Fang ZY, Xu J et al. The epidemiology and burden of rotavirus in China: a review of the literature from 1983 to 2005. Vaccine 2007; 25: 406–413. 16956700 10.1016/j.vaccine.2006.07.054

[bib10] 10Sun LW, Tong ZL, Li LH et al. [Surveillance finding on rotavirus in Changchun children's hospital during July 1998–June 2001.] Zhong Hua Liu Xing Bing Xue Za Zhi 2003; 24: 1010–1012. Chinese. 14687501

[bib11] 11Tong ZL, Ma L, Zhang J et al. [Epidemiological study of rotavirus diarrhea in Beijing, China: a hospital-based surveillance from 1998–2001.] Zhong Hua Liu Xing Bing Xue Za Zhi 2003;24:1100–1103. Chinese. 14761624

[bib12] 12Cunliffe NA, Bresee JS, Hart CA. Rotavirus vaccines: development, current issues and future prospects. J Infect 2002; 45: 1–9. 12217724 10.1053/jinf.2002.1012

[bib13] 13World Health Organization. Report of the meeting on future directions for rotavirus vaccine research in developing countries. Geneva: WHO, 2000. Available at http://www.who.int/iris/handle/10665/66497 (accessed 6 September 2015).

[bib14] 14Fu C, He Q, Xu J et al. Effectiveness of the Lanzhou lamb rotavirus vaccine against gastroenteritis among children. Vaccine 2012; 31: 154–158. 23127516 10.1016/j.vaccine.2012.10.078

[bib15] 15Fu C, Tate JE, Jiang B. Effectiveness of Lanzhou lamb rotavirus vaccine against hospitalized gastroenteritis: further analysis and update. Hum Vaccine 2010; 6: 953. 10.4161/hv.6.11.12847 20980802

[bib16] 16Fu C, Wang M, Liang J et al. Effectiveness of Lanzhou lamb rotavirus vaccine against rotavirus gastroenteritis requiring hospitalization: a matched case-control study. Vaccine 2007; 25: 8756–8761. 18023510 10.1016/j.vaccine.2007.10.036

[bib17] 17Clemens JD, Kotloff KL, Kay B. Generic protocol to estimate the burden of Shigella diarrhoea and dysenteric mortality. Geneva: WHO, 1999. Available at http://www.who.int/iris/handle/10665/66151 (accessed 6 September 2015).

[bib18] 18World Health Organization. Generic protocols (i) hospital-based surveillance to estimate the burden of rotavirus gastroenteritis in children and (ii) a community-based survey on utilization of health care services for gastroenteritis in children. In: Biologicals Va, editor. Geneva: WHO, 2002. Available at http://www.who.int/immunization/diseases/rotavirus/generic_protocols/en/ (accessed 6 September 2015).

[bib19] 19Simmonds MK, Armah G, Asmah R et al. New oligonucleotide primers for P-typing of rotavirus strains: strategies for typing previously untypeable strains. J Clin Virol 2008; 42: 368–373. 18378188 10.1016/j.jcv.2008.02.011

[bib20] 20Jiang X, Huang PW, Zhong WM et al. Design and evaluation of a primer pair that detects both Norwalk- and Sapporo-like caliciviruses by RT-PCR. J Virol Methods 1999; 83: 145–154. 10598092 10.1016/s0166-0934(99)00114-7

[bib21] 21Ruuska T, Vesikari T. Rotavirus disease in Finnish children: use of numerical scores for clinical severity of diarrhoeal episodes. Scand J Infect Dis 1990; 22: 259–267. 2371542 10.3109/00365549009027046

[bib22] 22Bai ZS, Chen DM, Shen S. [Selection and characterization of strain LLR- 85 for oral rotavirus live vaccine.] Zhong Guo Sheng Wu Zhi Ping Xue Za Zi 1994; 7: 46–52. Chinese.

[bib23] 23Clark HF, Offit PA, Parashar UD et al. Rotavirus vaccines. In: Plotkin SA, Orenstein WA, Offit PA, editors. Vaccines, 5th edn. Atlanta: Elsevier Inc., 2008: 715–734.

[bib24] 24Orenstein WA, Bernier RH, Dondero TJ et al. Field evaluation of vaccine efficacy. Bull World Health Organ 1985; 63: 1055–1068. 3879673 PMC2536484

[bib25] 25Shapiro ED. Case-control studies of the effectiveness of vaccines: validity and assessment of potential bias. Pediatr Infect Dis J 2004; 23: 127–131. 14872178 10.1097/01.inf.0000109248.32907.1d

[bib26] 26Schlesselman JJ. Case-control studies: design, conduct, analysis. New York: Oxford University Press, 1982.

[bib27] 27Vesikari T, Isolauri E, D'Hondt E et al. Protection of infants against rotavirus diarrhoea by RIT 4237 attenuated bovine rotavirus strain vaccine. Lancet 1984; 1: 977–981. 6143964 10.1016/s0140-6736(84)92323-7

[bib28] 28Parashar UD, Steele D, Neuzil K et al. Progress with rotavirus vaccines: summary of the Tenth International Rotavirus Symposium. Expert Rev Vaccines 2013; 12: 113–117. 23414403 10.1586/erv.12.148

[bib29] 29Ruiz-Palacios GM, Pérez-Schael I, Velázquez FR et al. Safety and efficacy of an attenuated vaccine against severe rotavirus gastroenteritis. N Engl J Med 2006; 354: 11–22. 16394298 10.1056/NEJMoa052434

[bib30] 30Vesikari T, Matson DO, Dennehy P et al. Safety and efficacy of a pentavalent human-bovine (WC3) reassortant rotavirus vaccine. N Engl J Med 2006; 354: 23–33. 16394299 10.1056/NEJMoa052664

[bib31] 31Bresee JS, Hummelman E, Nelson EA et al. Rotavirus in Asia: the value of surveillance for informing decisions about the introduction of new vaccines. J Infect Dis 2005; 192: S1–S5. 16088790 10.1086/431515

[bib32] 32Clemens J, Brenner R, Rao M et al. Evaluating new vaccines for developing countries. Efficacy or effectiveness? JAMA 1996; 275: 390–397. 8569019

[bib33] 33Armah GE, Sow SO, Breiman RF et al. Efficacy of pentavalent rotavirus vaccine against severe rotavirus gastroenteritis in infants in developing countries in sub-Saharan Africa: a randomised, double-blind, placebo-controlled trial. Lancet 2010; 376: 606–614. 20692030 10.1016/S0140-6736(10)60889-6

[bib34] 34Zaman K, Dang DA, Victor JC et al. Efficacy of pentavalent rotavirus vaccine against severe rotavirus gastroenteritis in infants in developing countries in Asia: a randomised, double-blind, placebo-controlled trial. Lancet 2010; 376: 615–623. 20692031 10.1016/S0140-6736(10)60755-6

[bib35] 35Soares-Weiser K, Maclehose H, Bergman H et al. Vaccines for preventing rotavirus diarrhoea: vaccines in use. Cochrane Database Syst Rev 2012; 11: CD008521. 23152260 10.1002/14651858.CD008521.pub3

[bib36] 36Matson DO, O'Ryan ML, Pickering LK et al. Characterization of serum antibody responses to natural rotavirus infections in children by VP7-specific epitope-blocking assays. J Clin Microbiol 1992; 30: 1056–1061. 1374761 10.1128/jcm.30.5.1056-1061.1992 PMC265223

[bib37] 37Ward RL, Clemens JD, Knowlton DR et al. Evidence that protection against rotavirus diarrhea after natural infection is not dependent on serotype-specific neutralizing antibody. J Infect Dis 1992; 166: 1251–1257. 1331249 10.1093/infdis/166.6.1251

[bib38] 38Li Y, Wang SM, Zhen SS et al. Diversity of rotavirus strains causing diarrhea in <5 years old Chinese children: a systematic review. PLoS One 2014; 9: e84699. 24416267 10.1371/journal.pone.0084699 PMC3885581

[bib39] 39Smith PG, Rodrigues LC, Fine PE. Assessment of the protective efficacy of vaccines against common diseases using case-control and cohort studies. Int J Epidemiol 1984; 13: 87–93. 6698708 10.1093/ije/13.1.87

[bib40] 40Orenstein WA, Bernier RH, Hinman AR. Assessing vaccine efficacy in the field. Further observations. Epidemiol Rev 1988; 10: 212–241. 3066628 10.1093/oxfordjournals.epirev.a036023

[bib41] 41Comstock GW. Evaluating vaccination effectiveness and vaccine efficacy by means of case-control studies. Epidemiol Rev 1994; 16: 77–89. 7925730 10.1093/oxfordjournals.epirev.a036147

[bib42] 42Zheng JS, Cao L, Guo SC et al. [Survey on the immunization status of category B vaccine among children aged 1 to 2 years in China.] Zhong Guo Yi Miao He Mian Yi 2012; 18: 233–237. Chinese.

[bib43] 43Wang ZB, Wang W, Guo WS et al. [Survey on vaccination rate of second class vaccine and influencial factors in children in rural areas of nine counties in Henan province.] Wei Sheng Wu Xue Mian Yi Xue Jin Zhan 2013; 41:53–61. Chinese.

[bib44] 44Chang J, Hou ZY, Yue DH et al. [Factors related to self-paid vaccination and its related factors among children aged 0-3 years in China.] Zhong Guo Gong Gong Wei Sheng 2014; 30: 579–582. Chinese.

